# Biopsy Provides Actionable Information Associated with Treatment Decisions for 2–7 cm Renal Masses

**DOI:** 10.3390/cancers18172859

**Published:** 2026-09-04

**Authors:** Daniel F. Roadman, Daniel D. Shapiro, Paz Lotan, Tudor Borza, Isabelle SueJin Lee, J. Louis Hinshaw, Fred T. Lee, Edwarda Golden, Erica Knavel Koepsel, Paul Laeseke, David F. Jarrard, Kyle A. Richards, Elizabeth L. Koehne, Glenn O. Allen, Michael C. Risk, E. Jason Abel

**Affiliations:** 1 Department of Urology, University of Wisconsin School of Medicine and Public Health, Madison, WI 53726, USA; roadman@urology.wisc.edu (D.F.R.); ddshapiro@wisc.edu (D.D.S.); pazlotan@gmail.com (P.L.); tborza@med.umich.edu (T.B.); islee@wisc.edu (I.S.L.); jarrard@urology.wisc.edu (D.F.J.); richardsk@urology.wisc.edu (K.A.R.); koehne@urology.wisc.edu (E.L.K.); alleng@urology.wisc.edu (G.O.A.); risk@urology.wisc.edu (M.C.R.); 2 Department of Urology, University of Michigan, Ann Arbor, MI 48109, USA; 3 Department of Radiology, University of Wisconsin School of Medicine and Public Health, Madison, WI 53726, USA; jlhinshaw@wisc.edu (J.L.H.); ftlee@wisc.edu (F.T.L.J.); edesouza@wisc.edu (E.G.); knavel@wisc.edu (E.K.K.); plaeseke@wisc.edu (P.L.)

**Keywords:** renal cell carcinoma, renal mass biopsy, active surveillance

## Abstract

**Background/Objectives**: Renal mass biopsy (biopsy) is increasingly incorporated into the evaluation of localized renal masses, yet its role in guiding management remains variable among clinical practices. We evaluated the clinical association of biopsy using a simplified classification system categorizing biopsy results as Biopsy Actionable for Immediate Treatment (BAIT) or Biopsy Actionable for Active Surveillance (BAAS)—assessing how biopsy-derived histology influences real-world management. **Methods**: We identified all patients undergoing biopsy for a renal mass measuring 2–7 cm at a single academic center between 2010 and 2025. After excluding nondiagnostic biopsies, repeat biopsies, and biopsies performed for metastatic diagnosis, 1395 patients formed the analytic cohort. Histologic subtypes and initial treatment were evaluated. Multivariable logistic regression assessed predictors of (1) radical versus partial nephrectomy, (2) surgery versus thermal ablation, and (3) active surveillance versus intervention, adjusting for tumor diameter, age, and BAIT/BAAS status. **Results**: Of 1395 patients, 1119 (80.2%) had BAIT pathology and 276 (19.8%) had BAAS pathology. Initial management included surgery (36.3%), thermal ablation (36.3%), active surveillance (24.2%), no treatment (1.7%), and radiation (1.5%). BAAS pathology was strongly associated with the selection of active surveillance (OR 21.80, *p* < 0.001). Larger tumor diameter was the principal driver of radical nephrectomy (OR 2.04/cm, *p* < 0.001) and of choosing surgery over ablation (OR 2.05/cm, *p* < 0.001). Younger age was associated with selection of surgery over ablation (OR 0.93/year, *p* < 0.001). Biopsy histology showed a stronger association with surveillance selection than either tumor diameter or age. **Conclusions**: Biopsy provides actionable diagnostic information for patients with 2–7 cm renal masses, identifying benign or indolent pathology in nearly one in five patients. BAIT/BAAS categorization was strongly associated with the selection of active surveillance for renal masses.

## Simple Summary

Patients with stage 1 non-metastatic renal masses often have a choice between partial nephrectomy, radical nephrectomy, thermal ablation, radiation, or active surveillance. Renal mass biopsy can be obtained to make a pathologic diagnosis, but it remains unclear how often biopsy diagnosis provides actionable information enabling treatment decisions. To investigate the association between biopsy and decision-making, we reviewed records from 1395 patients who had a renal mass biopsy for renal masses measuring 2 to 7 cm over 15 years. Among patients with a diagnostic biopsy, 19.8% had benign or indolent pathology considered compatible with active surveillance, and three-quarters of these patients were managed with active surveillance rather than treatment. These findings support wider use of renal mass biopsy to guide safer, more personalized care.

## 1. Introduction

Evaluation and treatment of localized renal masses have evolved substantially over the past two decades. Improved cross-sectional imaging has increased incidental detection and the recognition of indolent tumor biology in many renal masses has expanded treatment options for patients [1]. Active surveillance (AS) is recommended by guidelines for renal masses < 2 cm [2]. With increasing tumor diameter, malignant potential increases, and larger masses present a particular management challenge. However, risk is variable among different types of tumors, and surgical series demonstrate that tumors ≤ 4 cm encompass a wide spectrum of pathologic risk, including approximately 20% benign tumors in addition to variable rates of high-grade or pT3 disease [3]. More recent work has extended these observations and reinforced that diameter alone is an imperfect surrogate for biologic behavior, supporting a more nuanced, risk-adapted approach to management [4,5]. Prospective AS data from the Delayed Intervention and Surveillance for Small Renal Masses Registry, including cohorts of younger patients, have further demonstrated that AS can be safely utilized even in highly selected younger individuals without compromising oncologic outcomes [6].

Renal mass biopsy (biopsy) has become an important tool in this context. Modern core-needle biopsy provides high diagnostic accuracy for distinguishing benign from malignant lesions, with meta-analyses reporting diagnostic rates of roughly 90% and pooled sensitivity and specificity in the mid- to high-90% range for malignancy detection [7,8]. Single-center and multicenter series have confirmed that biopsy results meaningfully influence management decisions and are particularly valuable in patients for whom AS or nephron-sparing options are being considered [9,10,11,12].

Multiple contemporary reviews now frame biopsy as a central component of risk stratification rather than a purely diagnostic exercise. Narrative and systematic reviews highlight the role of biopsy in reducing the overtreatment of benign or indolent lesions, supporting patient-centered decision-making and informing choices among surgery, thermal ablation (TA), and AS [8,13,14]. Other authors emphasize that biopsy use has expanded alongside AS and TA strategies but remains underutilized and heterogeneous across practice settings, particularly for intermediate-diameter tumors [15,16,17].

To investigate the utility of biopsy in pragmatic terms, we can broadly separate results into categories favoring initial treatment or surveillance: Biopsy Actionable for Immediate Treatment (BAIT) and Biopsy Actionable for Active Surveillance (BAAS). As such, this study investigates the association of biopsy results among other factors (age, tumor diameter) associated with initial treatment selection of active surveillance (AS), thermal ablation (TA), partial nephrectomy (PN), or radical nephrectomy (RN).

## 2. Materials and Methods

All patients at a high-volume academic medical center who underwent percutaneous biopsy between January 2010 and November 2025 were identified from a prospectively maintained institutional database. Demographic, clinical, radiologic, procedural, and pathologic data were analyzed. All biopsies were interpreted by genitourinary pathologists using standardized reporting templates. Institutional review board approval was obtained.

Eligible patients had a radiographically measured non-metastatic solid renal mass between 2 and 7 cm at the time of biopsy and underwent an initial diagnostic biopsy during the study period. Exclusion criteria included nondiagnostic biopsies [18], repeat biopsies of the same renal mass, and biopsies obtained solely for staging or confirmation of metastatic renal cell carcinoma rather than for the local management of the primary tumor. Biopsy was offered at the discretion of the attending physician when it could impact management selection for AS, TA, PN, or RN [2,19]. Prior studies at our institution noted a 42% biopsy utilization for patients with renal masses 4 cm or less [20].

Biopsies always preceded treatment so that biopsy information was available and discussed with patients prior to treatment selection. Biopsies were performed under computed tomography or ultrasound guidance at the discretion of the radiologist. Procedural variables included the number of cores obtained, needle gauge, and the use of quadrant sampling. Pathologic assessment included the assignment of the histologic subtype, nuclear grade when applicable, and sarcomatoid or rhabdoid differentiation when present.

Biopsy histology was categorized into two groups based on whether treatment of the primary tumor histology would be recommended for most patients. The first group was named the “Biopsy Actionable for Immediate Treatment” (BAIT) which was defined as patients with a histologic diagnosis of renal cell carcinoma (RCC) or another malignancy. Given that all patients within our cohort had tumors ≥ 2 cm, we considered patients diagnosed with RCC or other non-systemic malignancies would be reasonable candidates for immediate treatment, consistent with guideline recommendations [2]. Patients within the BAIT category may have ultimately elected for surveillance rather than immediate treatment after shared decision-making. The second group was named the “Biopsy Actionable for Active Surveillance” (BAAS) group, which was defined as a histologic diagnosis of benign or indolent neoplasms considered appropriate for active surveillance [21,22,23]. While the majority of patients were readily classified using this framework, several individual histologic subtypes required additional clarification. Within BAAS, oncocytoma, low-grade oncocytic tumor, hybrid oncocytic chromophobe tumor, and angiomyolipoma were classified as benign or indolent per current World Health Organization (WHO) terminology [24]. Clear cell papillary renal cell tumor, reclassified from “carcinoma” to “tumor” in the 2022 WHO classification given its indolent behavior, was classified as BAIT here, reflecting management patterns in place before this reclassification, which postdates most of our study period. Lymphoma was classified as BAAS given its systemic, non-surgical management. Given the rarity of some individual subtypes, classifications of less common entities should be interpreted with caution. Patients within the BAAS category may have elected to undergo immediate treatment after shared decision-making regardless of BAAS categorization. Initial management following biopsy was classified as surgery (PN or RN), TA, AS, locoregional radiotherapy (RT), or no local treatment or palliative care only.

The primary outcomes of interest were patterns of initial management after biopsy and predictors of treatment selection. Univariable comparisons between BAIT and BAAS cohorts used the Wilcoxon rank-sum test for continuous variables and Fisher’s exact test for categorical variables. Multivariable logistic regression models were constructed to evaluate three comparisons: (1) RN versus PN among patients who underwent surgery; (2) surgery (RN or PN) versus TA; and (3) AS versus any intervention (PN, RN, TA). Covariates included radiographic tumor diameter, age at biopsy, and BAIT/BAAS category. Patients receiving RT (*n* = 21) and no-treatment/palliative treatment (*n* = 24) were excluded from this specific model. Odds ratios (ORs) with 95% confidence intervals (CIs) were estimated. For all statistical tests, a *p*–value of less than 0.05 was considered statistically significant.

## 3. Results

A total of 1395 patients with 2–7 cm renal masses met inclusion criteria (Figure S1). The median age at biopsy was 67 years (interquartile range [IQR] 59–73), and the median tumor diameter was 3.3 cm (IQR 2.5–4.2) (Table 1). Clavien–Dindo [25] grade 3 or higher complications occurred in three (0.2%) patients. Histologic findings categorized 1119 patients (80.2%) as BAIT and 276 (19.8%) as BAAS (Table 2). Within the BAIT group, pathology was predominantly composed of clear cell RCC (810 cases), papillary RCC (182 cases), and chromophobe RCC (46 cases), with smaller numbers of other malignant subtypes. BAAS pathology primarily consisted of oncocytoma (137 cases), low-grade oncocytic tumors (99 cases), and angiomyolipoma (31 cases). BAAS lesions were more common among smaller tumors but remained present across the full-diameter range, including 13.1% of all 4–7 cm tumors (Table S1).

**Figure 1:**
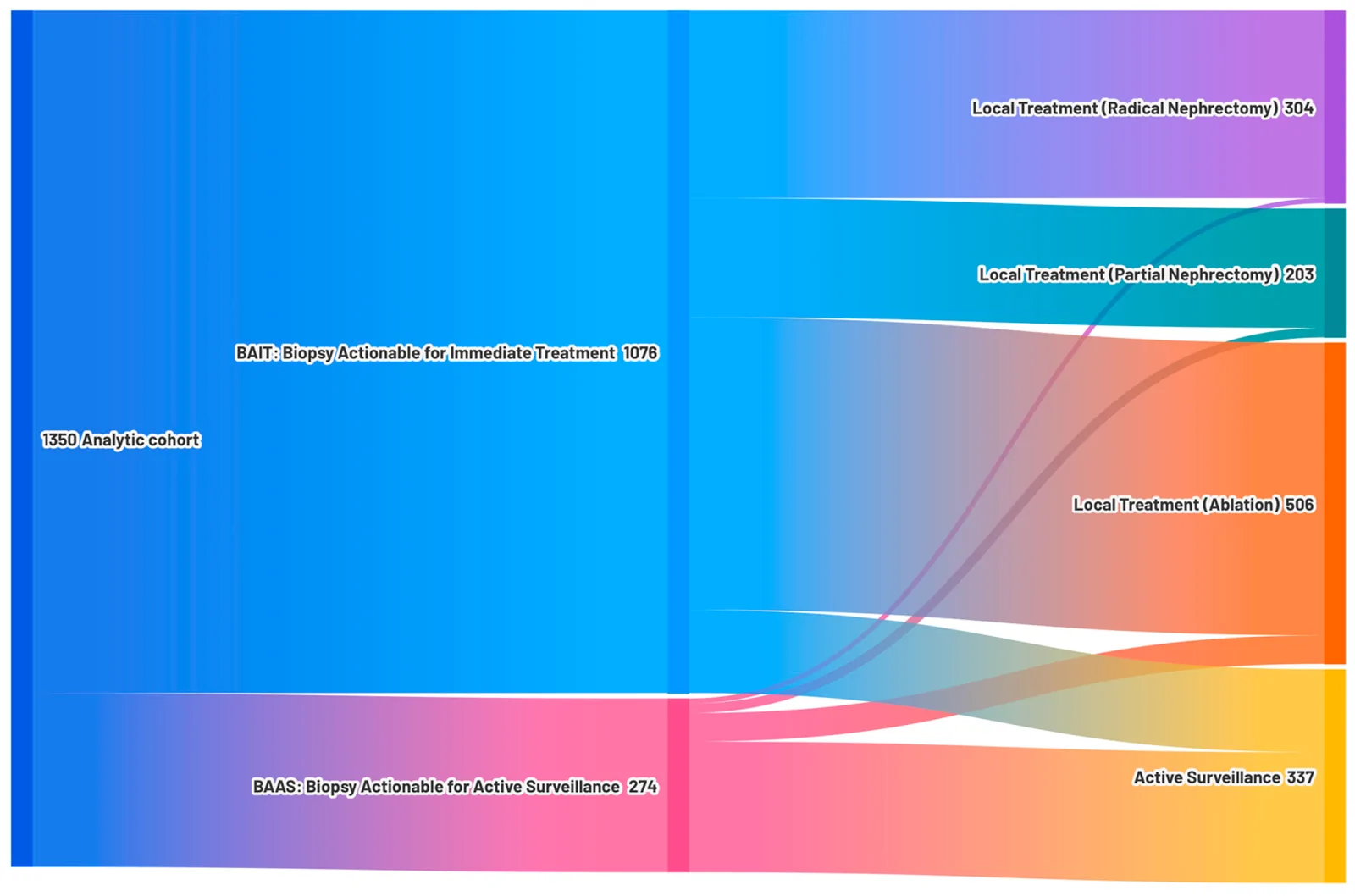
Management pathways for local treatment following renal mass biopsy categorization: Biopsy Actionable for Immediate Treatment (BAIT)–Biopsy Actionable for Active Surveillance (BAAS). N = 1350 as no treatment/palliative (*n* = 24) and radiation (*n* = 21) were removed for clarity.

Treatment selection across the cohort demonstrated substantial heterogeneity and was influenced by BAIT/BAAS classification (Figure 1). Overall, 507 (36.3%) patients underwent surgery, 506 (36.3%) received TA, and 337 (24.2%) were managed with AS. BAIT/BAAS classification demonstrated the strongest association with AS selection: 74.6% of BAAS lesions compared with only 11.7% of BAIT lesions. In multivariable modeling, patients with BAAS pathology had nearly 22-fold higher odds of observation than treatment (OR 21.80, 95% CI 15.40–30.87; *p* < 0.001) (Table 3). Older age was associated with increased odds of AS (OR 1.06 per year 95% CI 1.05–1.08; *p* < 0.001), whereas increasing tumor diameter was associated with lower odds of AS (OR 0.85 per cm 95% CI 0.75–0.98; *p* = 0.02).

Among 507 patients undergoing RN or PN, tumor diameter independently predicted RN versus PN (OR 2.04 per cm 95% CI 1.72–2.41; *p* < 0.001), and older age similarly favored RN (OR 1.04 per year 95% CI 1.02–1.06; *p* < 0.001), while BAIT/BAAS status was not independently associated with RN and PN (*p* = 0.06).

In analyses comparing surgery with TA (*n* = 1013), tumor diameter was the dominant predictor of surgical management (OR 2.05 per cm 95% CI 1.81–2.33; *p* < 0.001), whereas increasing age favored TA over surgery (OR 0.93 per year 95% CI 0.91–0.94; *p* < 0.001). BAAS categorization was associated with lower odds of undergoing surgery instead of thermal ablation (OR 0.42, 95% CI 0.23–0.77; *p* = 0.01).

## 4. Discussion

This study demonstrates the association between biopsy and treatment decision-making for 2–7 cm renal masses, using a straightforward framework separating biopsy results favoring kidney mass treatments or surveillance. Among 1395 patients, biopsy-derived histology was a strong independent predictor of choosing surveillance, adjusting for both age and tumor diameter. Patients with pathology actionable for surveillance represented 20% of the cohort and had nearly 22 times higher odds of being managed with imaging surveillance. Similarly, biopsy results actionable for surveillance were an important predictor for patients choosing TA vs. surgery, in addition to age and tumor diameter. However, among patients treated surgically, biopsy results were not significantly associated with choosing RN vs. PN, after adjusting for age and tumor diameter. Taken together, these data provide evidence for how renal mass pathologic diagnosis influences management, providing actionable information to patients and enabling many patients to avoid local interventions.

These findings extend and operationalize a conceptual framework articulated in several contemporary reviews. Previously, studies have argued that the principal value of biopsy lies in its ability to refine risk stratification and support shared decision-making, particularly when the spectrum of possible diagnoses ranges from benign to highly aggressive disease [13,14]. Similarly, others emphasized that biopsy should be viewed as a management tool rather than a purely diagnostic test, with the potential to avoid unnecessary nephrectomy and to direct patients toward AS or minimally invasive options when biologically appropriate [15,16,17]. In practice, the BAIT/BAAS framework can be communicated to patients as a simple treat now versus observe dichotomy grounded in histology, which may facilitate shared decision-making for intermediate-sized tumors where defaulting to intervention has historically been common. The BAIT/BAAS system offers a pragmatic way to translate complex pathology reports into management-relevant categories. Previous studies identified safety for observation in the BAAS categories. For example, patients with renal oncocytic neoplasms managed with surgery, TA, or AS had excellent cancer-specific outcomes, with no progression in carefully selected AS cohorts [26]. Others have described similarly indolent behavior for small renal masses under AS, particularly in older or comorbid patients, with low rates of metastatic progression when appropriate selection and follow-up protocols are used [4,5]. By obtaining a biopsy and aggregating such lesions into an actionable for surveillance category, the present study provides a clinically intuitive pathway for confidently recommending AS when histology supports a low-risk phenotype and patient factors favor non-operative management.

At the same time, our analysis underscores that biopsy provides biologic information that complements rather than replaces anatomic and technical considerations. Decisions regarding PN versus RN remain closely linked to baseline renal function, tumor diameter, location, and complexity, as reflected in tools such as the R.E.N.A.L. nephrometry score and in broader surgical literature demonstrating increased ischemia time, complication risk, and conversion to RN with more complex tumors [27,28,29]. Similarly, the preference for TA over surgery in older patients in our cohort mirrors contemporary data demonstrating that TA can offer acceptable oncologic control with lower perioperative morbidity in selected patients, albeit with shorter follow-up and less robust long-term data than surgery [30,31]. By clarifying whether a lesion is likely to be benign, indolent, or clearly malignant, biopsy helps position patients along this continuum, but it complements rather than replaces considerations of surgical complexity, renal function, and patient preference.

Our cohort, spanning 2–7 cm, includes both cT1a (≤4 cm) and cT1b (>4–7 cm) tumors. BAAS pathology was proportionally more common among smaller masses within our cohort, comprising 53.2% of the 2.0–2.9 cm masses compared with 13.1% of the 4.0–7.0 cm masses, consistent with the well-established inverse relationship between tumor size and the probability of aggressive or malignant histology [32]. This pattern supports the biological plausibility of extending biopsy-informed decision-making to cT1b masses, since a meaningful proportion of larger lesions in our cohort still yielded pathology compatible with active surveillance. Size continues to carry management implications even within BAAS, however: AML lesions exceeding 4 cm carry a meaningful rupture risk and may warrant intervention regardless of BAAS classification [23]. A separate, histology-based nuance also applies within BAAS, as oncocytoma cannot always be distinguished from chromophobe RCC on core biopsy alone, though concordance between biopsy and final nephrectomy pathology for oncocytic renal neoplasms remains high (93%) [33]. Taken together, these findings indicate that while individual entities within BAIT/BAAS may still require case-by-case judgment, similar to the judgment already involved in deciding whether to pursue biopsy at all, the framework reliably separates the large majority of patients into clear intervention-versus-surveillance groups across the size and histologic spectrum captured by biopsy.

Safety considerations remain central to decisions about when to perform biopsy. Large contemporary analyses report low overall complication rates for biopsy, with most events consisting of self-limited bleeding or pain and major complications occurring in well under 1% of cases [8,18]. The latter study, which evaluated 1155 consecutive core biopsies, found that tumor diameter, cystic architecture, and fewer cores were associated with nondiagnostic sampling but did not identify modifiable predictors of serious complications [18]. Case reports and reviews continue to describe needle-track seeding as a very uncommon event, particularly in older series or in settings where large cutting needles or open biopsies were used [15,34]. We did not experience any needle-tract seeding in our cohort, and major Clavien–Dindo complications were rare (0.2%). Overall, when biopsy is performed using guideline-based techniques and periprocedural management, whether via a coaxial or direct puncture approach, the balance of evidence supports a favorable safety profile in appropriately selected patients [12,13,18,35].

Limitations that should guide interpretation in this study include retrospective, single-center design that introduces the potential for selection bias in both the decision to pursue biopsy and subsequent management choices, and findings should not be generalized to all non-biopsied patients. Biopsy practice patterns may differ in community settings or centers with less availability to provide biopsies. Nondiagnostic biopsies are more common among smaller and cystic renal masses [36], and their exclusion may bias our cohort toward lesions with more definitive histology, potentially inflating the apparent discriminative value of biopsy relative to its performance across the full range of biopsied 2–7 cm masses. The BAIT/BAAS framework depends on accurate and detailed pathology analysis and may group together entities with heterogeneous biology, particularly within the BAIT category, and our models did not adjust for comorbidity burden, renal function, or tumor complexity, which may also influence treatment selection. BAIT/BAAS classification was strongly associated with but did not uniformly dictate treatment selection: 11.7% of BAIT patients nonetheless selected AS, and 8.3% of BAAS patients underwent surgery. Crossover to surgery among BAAS patients was concentrated among oncocytic and chromophobe renal tumors, consistent with the diagnostic uncertainty in distinguishing these lesions from chromophobe RCC on core biopsy. Interval growth or patient preference may also have influenced the decision to proceed with treatment despite a BAAS-qualifying histology. Finally, the focus of this study was to evaluate the association between biopsy and initial treatment decision, and we did not evaluate long-term outcomes according to biopsy status, and some patients may be treated after initial surveillance as seen in other studies [37]. Despite these limitations, this study adds meaningful evidence that biopsy is associated with real-world treatment decisions for cT1 renal masses. By demonstrating that BAIT/BAAS categorization is a dominant predictor of AS, while tumor diameter and age govern the choice among surgical and ablative modalities, our findings illustrate how biologic and anatomic information can be integrated to support individualized care. Biopsy-derived histologic information may also hold value beyond initial management selection, as ongoing work continues to identify treatment-related biomarkers and mechanisms of resistance relevant to systemic therapy in RCC [38,39,40]. In an era where surveillance and TA are increasingly utilized in selected patients, incorporating biopsy into the routine evaluation of 2–7 cm renal masses offers a rational path toward more precise, patient-centered management.

## 5. Conclusions

Biopsy provides actionable diagnostic information for 2–7 cm renal masses and exerts a substantial influence on real-world treatment decisions. Biopsy classification showed the strongest association with active surveillance as initial treatment, surpassing traditional clinical predictors such as tumor diameter and patient age. As the future integrates advanced imaging biomarkers or molecular techniques, pathologic diagnosis with biopsy will be important for biologically informed, individualized treatment planning for renal tumors.

## Figures and Tables

**Table 1:** Clinical and pathologic variables of the study population.

Clinical and Pathologic Variables	Overall (N = 1395)	BAIT (N = 1119)	BAAS (N = 276)	*p*–Value
**Age at Biopsy (Years)**	67 [59–73]	66 [58–72.5]	69 [63–75]	<0.001
**Largest Radiographic Tumor Diameter (cm)**	3.3 [2.5–4.2]	3.4 [2.6–4.4]	3 [2.5–3.8]	<0.001
**Male Gender**	932 (66.8%)	756 (67.6%)	176 (63.8%)	0.25
**Number of Biopsy cores**				0.29
**1**	221 (15.8%)	183 (16.4%)	38 (13.8%)	
**2**	583 (41.8%)	456 (40.8%)	127 (46.0%)	
**3**	364 (26.1%)	290 (25.9%)	74 (26.8%)	
**4**	184 (13.2%)	152 (13.6%)	32 (11.6%)	
**≥5**	43 (3.1%)	38 (3.4%)	5 (1.8%)	
**Imaging used for Biopsy**				0.14
**US**	1303(93.4%)	1051 (93.9%)	252 (91.3%)	
**CT**	92 (6.6%)	68 (6.1%)	24 (8.7%)	
**Year of Biopsy**				0.48
**2010–2015**	332 (23.8%)	269 (24.0%)	63 (22.8%)	
**2016–2020**	443 (31.8%)	347 (31.0%)	96 (34.8%)	
**2021–2025**	620 (44.4%)	503 (45.0%)	117 (42.4%)	
**Nuclear Grade (Clear Cell and Papillary Only N = 992)**				1.0
**Grade 1–2**	836 (84.3%)	836 (84.3%)	0 (—)	
**Grade 3–4**	99 (10.0%)	99 (10.0%)	0 (—)	
**Not Graded**	57 (5.7%)	57 (5.7%)	0 (—)	
**Sarcomatoid Features**	5 (0.4%)	5 (0.4%)	0 (—)	—
**Rhabdoid Features**	17 (1.2%)	17 (1.5%)	0 (—)	—
**Clavien–Dindo Complication**				1.0
**Grade 1–2**	9 (0.6%)	7 (0.6%)	2 (0.7%)	
**Grade 3–4**	3 (0.2%)	2 (0.2%)	1 (0.4%)	
**Side of Tumor**				0.17
**Right**	730 (52.3%)	583 (52.1%)	147 (53.3%)	
**Left**	651 (46.7%)	522 (46.6%)	129 (46.7%)	
**Transplant**	14 (1.0%)	14 (1.3%)	0 (0.0%)	
**Initial Treatment**				<0.001
**Radical Nephrectomy**	304 (21.8%)	296 (26.5%)	8 (2.9%)	
**Partial Nephrectomy**	203 (14.6%)	188 (16.8%)	15 (5.4%)	
**Thermal Ablation**	506 (36.3%)	461 (41.2%)	45 (16.3%)	
**Active Surveillance**	337 (24.2%)	131 (11.7%)	206 (74.6%)	
**No Treatment/Palliative**	24 (1.7%)	22 (2.0%)	2 (0.7%)	
**Radiation**	21 (1.5%)	21 (1.9%)	0 (0.0%)	

**Table 2:** Distribution of biopsy results for 2–7 cm renal masses.

	*n* (%)	Included Biopsy Result	*n* (%)
**BAIT** Biopsy Actionable for Immediate Treatment	1119 (80.2%)	Clear cell RCC	810 (72.4%)
		Papillary RCC	182 (16.3%)
		Chromophobe RCC	46 (4.1%)
		RCC/Unspecified carcinoma	36 (3.2%)
		Clear cell papillary renal cell tumor	22 (2.0%)
		Urothelial carcinoma	7 (0.6%)
		Hybrid oncocytic chromophobe tumor	4 (0.4%)
		Sarcoma	3 (0.3%)
		Cystic RCC	3 (0.3%)
		Xanthogranulomatous pyelonephritis (XGP)	2 (0.2%)
		Translocation RCC	2 (0.2%)
		Collecting duct carcinoma	1 (0.1%)
		Juxtaglomerular (JG) tumor	1 (0.1%)
**BAAS** Biopsy Actionable for Active Surveillance	276 (19.8%)	Oncocytoma	137 (49.6%)
		Low–grade oncocytic tumor	99 (35.9%)
		Angiomyolipoma (AML)	31 (11.2%)
		Metanephric adenoma	6 (2.2%)
		Myxoid spindle cell tumor of the fat	2 (0.7%)
		Lymphoma/Leukemia	1 (0.4%)

**Table 3:** Multivariable predictors of treatment selection for 2–7 cm renal masses.

Predictors	Univariable		Multivariable	
	OR (95% CI)	*p*–Value	OR (95% CI)	*p*–Value
AS vs. Any Intervention (N = 1350)				
**BAAS vs. BAIT**	21.85 (15.72–30.38)	<0.001	21.80 (15.40–30.87)	<0.001
**Age (per year)**	1.06 (1.05–1.07)	<0.001	1.06 (1.05–1.08)	<0.001
**Tumor diameter (per cm)**	0.82 (0.74–0.92)	<0.001	0.85 (0.75–0.98)	0.02
RN vs. PN (N = 507)				
**BAAS vs. BAIT**	0.41 (0.17–0.97)	0.043	0.39 (0.15–1.02)	0.06
**Age (per year)**	1.05 (1.03–1.06)	<0.001	1.04 (1.02–1.06)	<0.001
**Tumor diameter (per cm)**	2.10 (1.78–2.48)	<0.001	2.04 (1.72–2.41)	<0.001
Surgery (RN/PN) vs. TA (N = 1013)				
**BAAS vs. BAIT**	0.49 (0.29–0.82)	0.006	0.42 (0.23–0.77)	0.01
**Age (per year)**	0.94 (0.93–0.96)	<0.001	0.93 (0.91–0.94)	<0.001
**Tumor diameter (per cm)**	1.83 (1.63–2.05)	<0.001	2.05 (1.81–2.33)	<0.001

## Data Availability

Data from this manuscript are available upon reasonable request from the corresponding author.

## Supplementary Materials

The following supporting information can be downloaded at: https://www.mdpi.com/article/10.3390/cancers18172859/s1, Figure S1. Study Selection Flow Diagram. Table S1. Distribution of Biopsy results Actionable for Immediate Treatment (BAIT) and Biopsy Results Categorized as Biopsy results Actionable for Active Surveillance (BAAS) across renal mass size categories 2–7 cm.

## Abbreviations

The following abbreviations are used in this manuscript:

BIOPSY	Renal Mass Biopsy
RCC	Renal Cell Carcinoma
AS	Active Surveillance
TA	Thermal Ablation
PN	Partial Nephrectomy
RN	Radical Nephrectomy
BAIT	Biopsy Actionable for Immediate Treatment
BAAS	Biopsy Actionable for Active Surveillance
