# Supplementary material for: Biopsy Provides Actionable Information Associated with Treatment Decisions for 2–7 cm Renal Masses
Source: Cancers (Basel). 2026 Sep 4;18(17):2859. doi: 10.3390/cancers18172859 (PMC13565656; doi:10.3390/cancers18172859)
Supplement: Supplementary file 1 [file cancers-18-02859-s001.zip › cancers-4543659-supplementary.pdf]

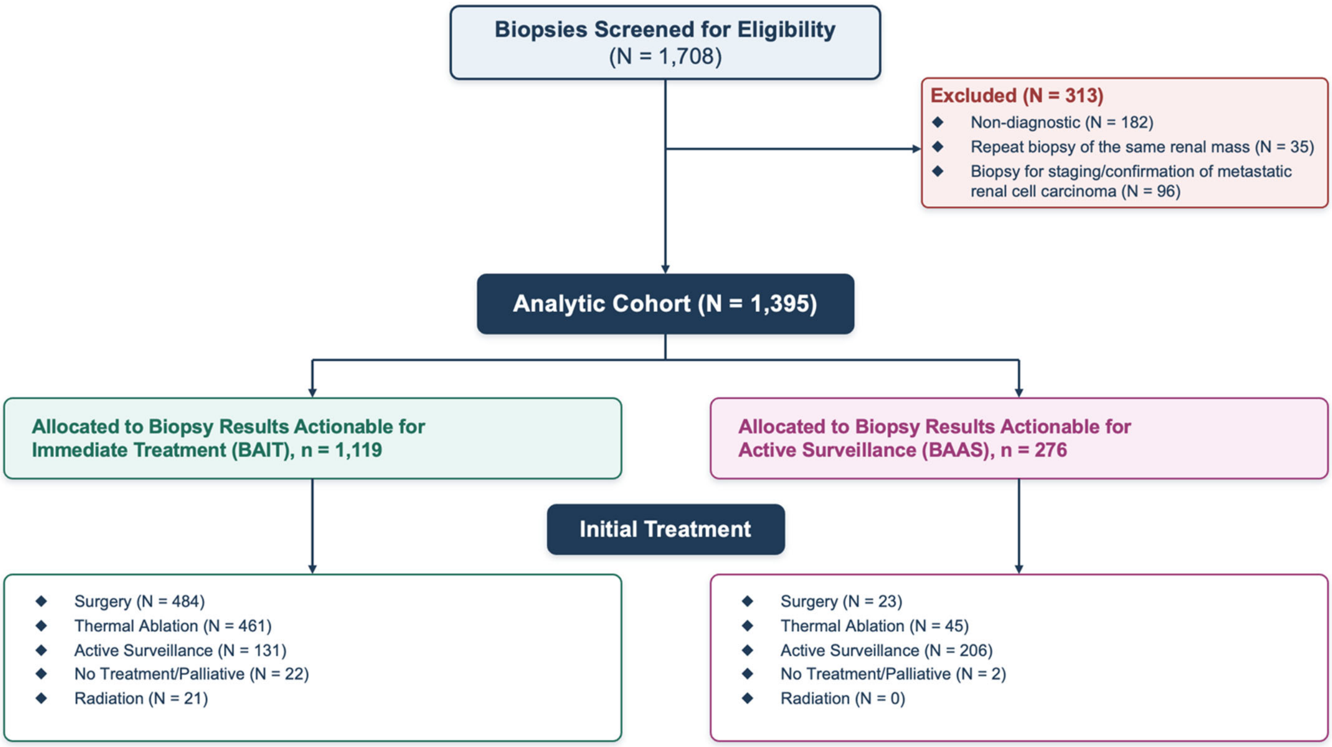

Figure S1. Study Selection Flow Diagram

Table S1. Distribution of Biopsy results Actionable for Immediate Treatment (BAIT) and Biopsy Results Categorized as Biopsy results Actionable for Active Surveillance (BAAS) across renal mass size categories 2–7 cm.

Renal Mass Size (cm)	BAIT (n = 1,119)*	BAAS (n = 276)	p-value
**2.0-2.9**	484 (43.3%)	147 (53.2%)	0.003
**3.0-3.9**	289 (25.8%)	77 (27.9%)	0.49
**4.0-4.9**	175 (15.6%)	29 (10.5%)	0.04
**5.0-5.9**	101 (9.0%)	17 (6.2%)	0.15
**6.0-7.0**	70 (6.3%)	6 (2.2%)	0.01

*Percentages reflect the proportion within each BAIT or BAAS column for that size category, not of the overall cohort.
